# Causal effects of gut microbiota on the risk of bipolar disorder: a Mendelian randomization study

**DOI:** 10.3389/frmbi.2023.1249518

**Published:** 2023-09-21

**Authors:** Ran Xu, Shuo Liu, Lu-yi Li, Ying Zhang, Guang-cheng Luo, Bo-qin Fang, Xin-jun Wang

**Affiliations:** ^1^ Department of Urology, Zhongshan Hospital Xiamen University, School of Medicine, Xiamen University, Xiamen, China; ^2^ Department of Urology, Zhongshan Hospital Xiamen University, The School of Clinical Medicine, Fujian Medical University, Xiamen, China

**Keywords:** gut microbiota, bipolar disorder, mendelian randomization, causal relationship, MiBioGen

## Abstract

**Background:**

Recent studies have suggested a possible association between gut microbiota and bipolar disorder (BD). However, observational studies are limited and there are variations between the gut microbiota taxa found in different studies. Therefore, we aimed to explore whether there is a causal relationship between gut microbiota and bipolar disorder at the genetic level and to reveal trends in the effect of influential gut microbiota on the development of bipolar disorder.

**Methods:**

We conducted a Mendelian randomisation (MR) study of summary statistics from a genome-wide association study (GWAS) of gut microbiota and bipolar disorder. Inverse variance weighting (IVW) was used as the primary method of statistical analysis, while results from the MR-Egger method, weighted median, weighted mode, and MR multiplicity residuals and outliers (MR-PRESSO) tests were used for additional validation.Cochrane’s Q test, MR-Egger intercept test, and MR-PRESSO global test were used to test MR results for stability and reliability.

**Result:**

We identified 13 gut microbial taxa causally associated with bipolar disorder. Betaproteobacteria, Acidaminococcaceae, Eubacterium xylanophilum group, Butyricimonas, Peptococcus, Prevotella 7, Roseburia, Terrisporobacter, Burkholderiales and Desulfovibrionales increased the risk of BD, whereas Candidatus Soleaferrea, Ruminiclostridium 5 and Victivallis decreased the risk of BD. The results of the MR analysis were shown to be reliable in the sensitivity analysis.

**Conclusion:**

With the MR study, we analysed the causal relationship between 196 gut microbial taxa and bipolar disorder and also identified gut microbiota associated with the risk of developing bipolar disorder. Our findings provide new biomarkers and potential therapeutic targets for the prevention and treatment of BD.

## 1 Introduction

Bipolar disorder (BD) is a psychiatric disorder characterised by alternating recurrent episodes of depression and mania, changes in activity levels and associated physical, psychological, cognitive and behavioural abnormalities.BD is a highly heterogeneous disorder of unknown aetiology (McIntyre et al., 2020). BD affects approximately 2% of the world’s population and represents a significant global public health burden (Lu et al., 2023). Although the disorder has been extensively studied from the perspectives of disease genetics, behaviour, physiopathology and imaging, no significant advances have been made (Sigitova et al., 2017; Vieta et al., 2018). Therefore, research into the aetiology of BD and the prevention of BD from its early onset has become increasingly important.

The gut microbiota is a collective of a large number of bacteria that live in the human gut. In recent years, a large body of research has demonstrated not only the important role of the gut microbiota in regulating metabolism and immune activity in the human body (Delzenne et al., 2011; Maynard et al., 2012; Lee et al., 2020), but also the close association with metabolic diseases, autoimmune diseases, tumours and many other diseases (Fan and Pedersen, 2021; Park et al., 2022; Miyauchi et al., 2023). The role of the gut microbiota in psychiatric disorders has also received increasing attention as the gut-brain axis continues to be studied (Nikolova et al., 2021). Compared to healthy individuals, there are significant changes in gut microbial composition or metabolic function in patients with BD (Painold et al., 2019). In addition, gut microbes are involved in the activation of focal inflammatory responses (Caruso et al., 2020), and similar inflammatory changes are significant in BD (Kirkpatrick and Miller, 2013). At the same time, gut microbes can directly or indirectly produce neurotransmitters such as GABA and 5-HT (Busnelli et al., 2019; Patterson et al., 2019), and the regulation of mood by these neurotransmitters has long been supported by previous studies (Mahar et al., 2014). These findings suggest a potential link between the gut microbiota and BD.

Mendelian randomization (MR) is a data analysis method for evaluating aetiological inference in epidemiological studies that uses genetic variation as an instrumental variable (IV) to estimate the causal relationship between the exposure factor of interest and the outcome of interest (Davies et al., 2018). MR uses Mendel’s first and second laws of inheritance, where parental alleles are randomly assigned to offspring, so that the relationship between genes and outcomes is not confounded by common confounders such as postnatal environment, socioeconomics and behavioural habits (Lawlor et al., 2008). MR can overcome the limitations of observational studies and is increasingly being used to study psychiatric disorders (Daghlas et al., 2021; Rosoff et al., 2021; Guo et al., 2022). Therefore, MR is an ideal technique to explore the causal relationship between gut microbiota and BD.

Therefore, based on summary statistics from a large genome-wide association study (GWAS) dataset, we used MR analysis to explore potential causal relationships between gut microbiota and BD, and to identify taxa with potential impact on BD.

## 2 Materials and methods

### 2.1 Study design

**Figure 1:**
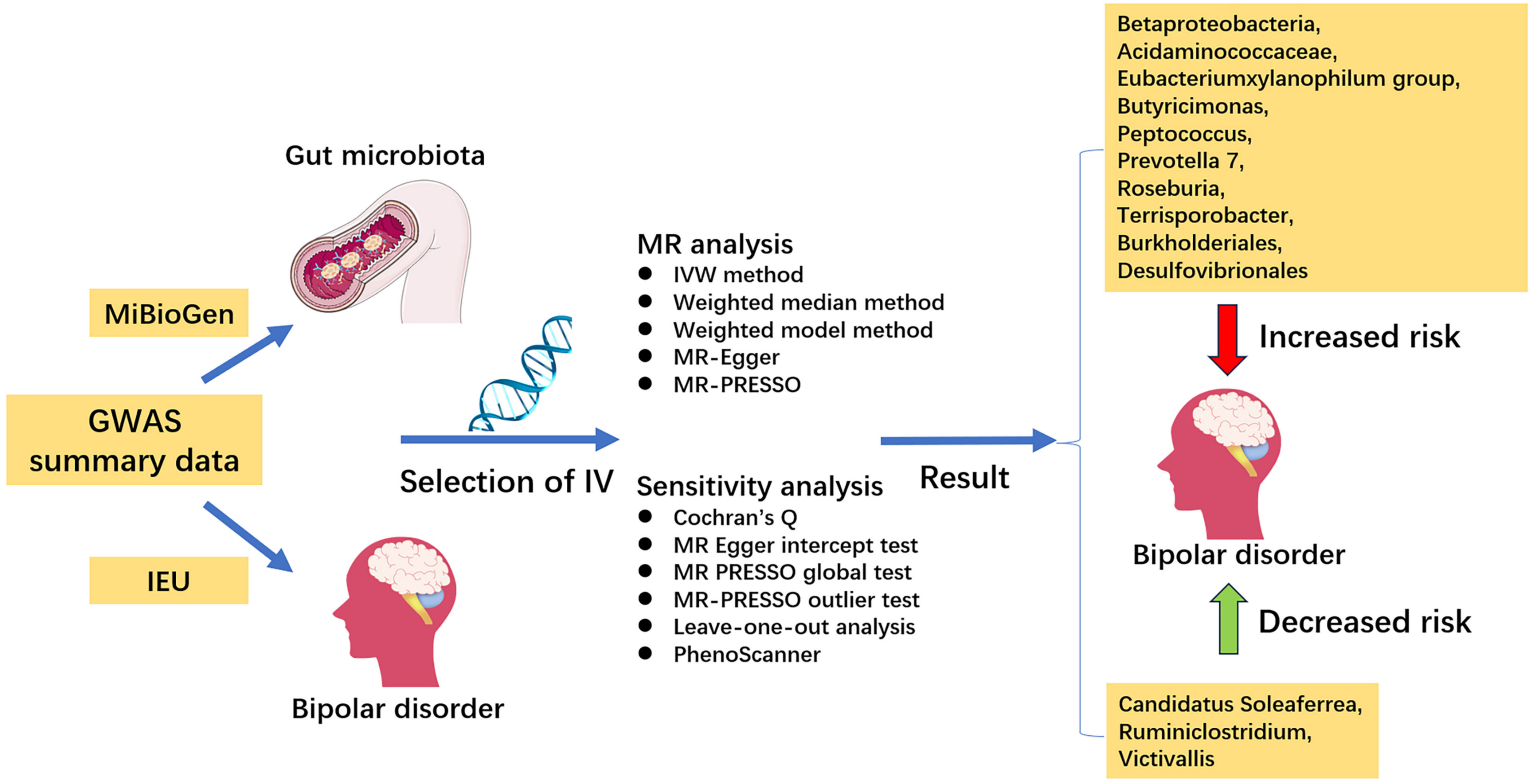
Overall design of this study.

The overall design of this study is shown in **Figure 1**. Mendelian randomisation studies must meet three core assumptions of correlation, independence and exclusivity, namely: (1) instrumental variables must be highly correlated with exposure factors, (2) instrumental variables must not be correlated with any confounding factors associated with ‘exposure-outcome’, and (3) instrumental variables can only influence outcome variables through exposure factors (Burgess et al., 2017).

### 2.2 Data sources

Genetic association data for the gut microbiota were obtained from a large GWAS study by the MiBioGen consortium (https://mibiogen.gcc.rug.nl), which included 18,340 participants from 11 countries. After removing 15 unknown bacterial taxa, we ended up with 9 phyla, 16 classes, 20 orders, 32 families and 119 genera, for a total of 196 taxa (**Table S1**) (Kurilshikov et al., 2021). GWAS summary statistics for BD were obtained from the IEU GWAS database (https://gwas.mrcieu.ac.uk/), a dataset (ieu-b-41) containing 20,352 cases of European ancestry and 31,358 controls of European ancestry. All patients with BD fulfilled international consensus criteria (DSM-IV or ICD-10) (Stahl et al., 2019).

### 2.3 Selection of instrumental variables

First, we extracted SNPs closely associated with gut microbiota from the GWAS study data using a threshold of P<1×10^-5^ (Zeng et al., 2023). In addition, to ensure the independence of each IV, we used a threshold of r^2^<0.001 and a window size of 10,000 kb to mitigate linkage disequilibrium (Zeng et al., 2023). At the same time, palindromic SNPs and SNPs not present in the results were also removed from the IVs. Finally, SNPs with an F-statistic <10 were removed to eliminate bias caused by weak instrumental variables in the results (Burgess and Thompson, 2011). F=β^2^ (exposure)/SE^2^ (exposure) was used to calculate the strength of the IV (Lawlor et al., 2008; Burgess et al., 2017; Zhang et al., 2022).

### 2.4 Mendelian randomization analysis and sensitivity analysis

Inverse-variance-weighted (IVW) method, weighted median method, weighted model, MR-Egger and Mendelian randomization pleiotropy residual sum and outlier (MR-PRESSO) test were used in this study for Mendelian randomization analysis. The IVW method uses Wald estimators and delta’s to compute ratio estimates for each SNP, and then combines estimates from each SNP to obtain primary causation estimates (Burgess et al., 2013). Compared with the other four methods, IVW provided more accurate effect estimates and was therefore chosen as the primary method of analysis, with the other methods used as supplementary validation of IVW (Bowden et al., 2016; Larsson et al., 2017; Yavorska and Burgess, 2017). The MR online power calculation tool (https://shiny.cnsgenomics.com/mRnd/) was used to calculate the statistical power of the causal effect estimates (Burgess, 2014).

In addition, as the Inverse Variance Weighted method may be subject to invalid instrument bias or pleiotropy, this study tested the validity and robustness of the IVW results through sensitivity analysis. Firstly, the potential heterogeneity of the MR analysis results was quantified and tested using Cochran’s Q. Secondly, the horizontal pleiotropy of the results was assessed using the MR Egger intercept test and the MR PRESSO global test, with p < 0.05 as statistically significant (Burgess and Thompson, 2017). By setting the number of distributions to 10,000, the MR-PRESSO outlier test was also used to adjust for horizontal pleiotropy by detecting and removing outliers (Verbanck et al., 2018). In parallel, we assessed the effect of abnormal SNPs on the results of Mendelian randomization using leave-one-out analysis. To further rule out confounding, instrumental variables with significant MR estimates related to risk factors for bipolar disorder (smoking, obesity, type 2 diabetes) were examined and excluded using the PhenoScanner, and re-tested to see if the causal effects remained significant (Kamat et al., 2019; Vermeulen et al., 2021; Miola et al., 2022).

### 2.5 Statistical analysis

To obtain more rigorous conclusions, we corrected the P-values using the Bonferroni method. Considering that each character level (phylum, class, order, family and genus) includes several gut microbial taxa, the corrected threshold for each character level should be 0.05/N, where N is the number of taxa included in that character level. Thus, the corrected thresholds for the phylum, class, order, family and genus levels are 5.56 × 10^-3^ (0.05/9), 3.13 × 10^-3^ (0.05/16), 2.50 × 10^-3^ (0.05/20), 1.56 × 10^-3^ (0.05/32) and 4.20 × 10^-4^ (0.05/119) respectively. MR results with p-values less than the Bonferroni correction threshold were considered significant. MR results with p-values < 0.05 were considered nominally significant (Liu et al., 2023; Luo et al., 2023). A p-value < 0.05 was considered statistically significant for sensitivity analysis in this study. The association between gut microbiota and bipolar disorder was expressed as an odds ratio (OR) with its 95% confidence interval (CI).

All of the above analyses were primarily performed using the Two-Sample-MR package (version 0.5.7) with R software (version 4.2.3).

## 3 Results

### 3.1 Details of IVs

Through rigorous screening in the previous section, 2527 SNPs were identified as instrumental variables. The phylum, class, order, family and genus levels of the gut microbiota contained 122, 221, 272, 432 and 1480 instrumental variables, respectively. Details of the IVs are provided in **Table S2**.

### 3.2 MR analysis

**Figure 2:**
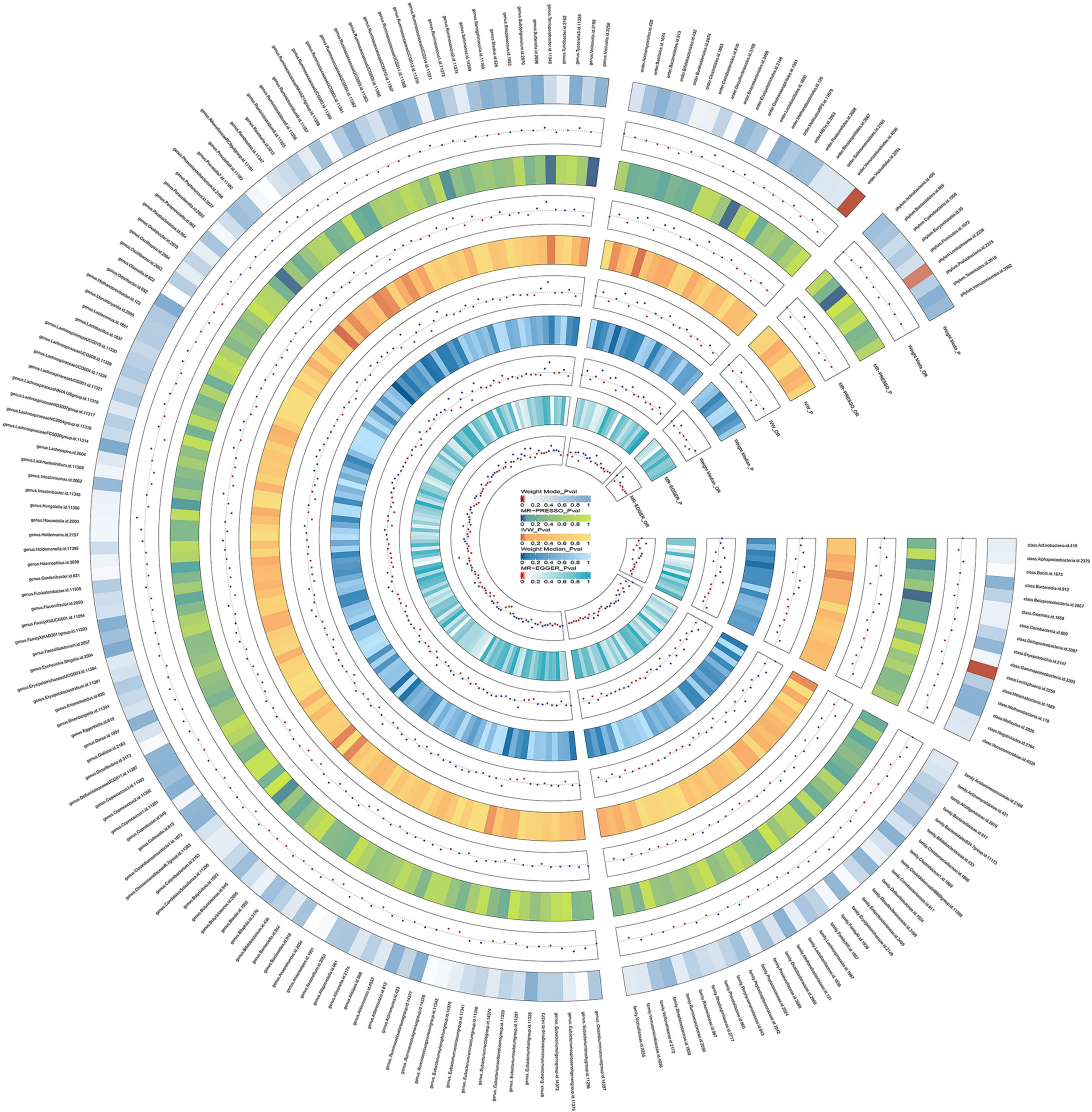
Preliminary MR estimates for the associations between gut microbiota and the risk of bipolar disorder. From the inner to outer circles, they represent the estimates of: MR-Egger, weighted median, inverse-variance weighted methods, MR-PRESSO and weighted mode, respectively. And the shades of color reflect the magnitude of the p-value.

The results of the preliminary analysis of the relationship between gut microbiota and bipolar disorder are shown in **Figure 2** and **Table S3**.

**Figure 3:**
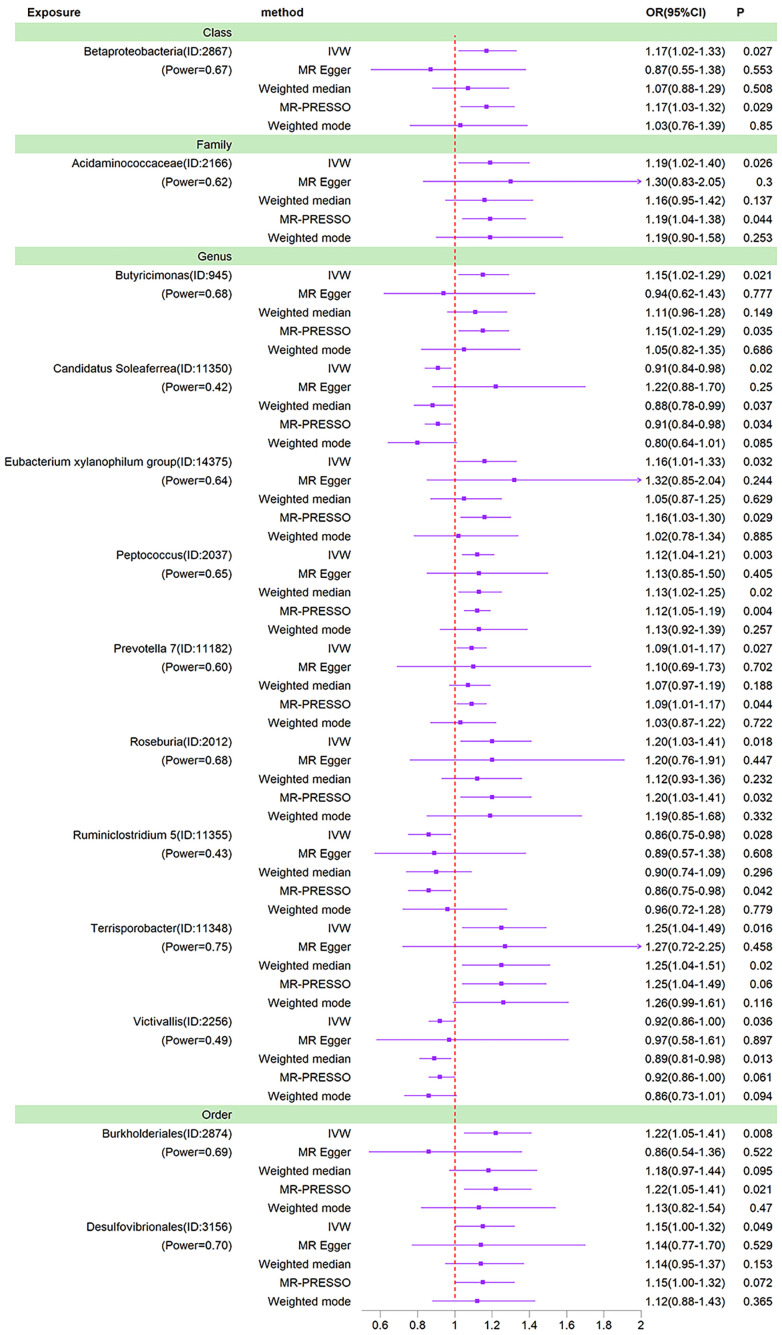
Forest plots of MR results for thirteen gut microbiota in bipolar disorder. Method, statistical analysis methods; OR, odds ratio; 95%CI, 95% confidence interval; P, significance P-value; Power, the statistical power of causal effect estimates.

We used IVW as the primary method for MR analysis of the 196 taxa tested. We detected a total of 16 taxa with P values < 0.05 for MR analysis results, and of these 16 taxa with nominal significance, we did not find any taxa with P values less than the Bonferroni correction threshold for MR analysis results. As shown in the sensitivity analysis below, the *Ruminococcaceae NK4A214 group* was found to be horizontally pleiotropic in the MR-Egger intercept test. The *Ruminococcaceae UCG-003* and *Bacillales* were found to be horizontally pleiotropic in the MR-PRESSO global test. These three taxa are therefore removed from the 16 taxa of nominal significance. So after removing these 3 taxa, we ended up with 13 nominally significant gut microbiota. *Prevotella 7* (OR=1.09, 95%CI=1.01-1.17, P=0.027), *Peptococcus* (OR=1.12, 95%CI=1.04-1.21, P=0.003), *Butyricimonas* (OR=1.15, 95%CI=1.02-1.29, P=0.021), *Desulfovibrionales* (OR=1.15, 95%CI=1.00-1.32, P=0.049), *Eubacterium xylanophilum group* (OR=1.16, 95%CI=1.01-1.33, P=0.032), *Betaproteobacteria* (OR=1.17, 95%CI=1.02-1.33, P=0.027), *Acidaminococcaceae* (OR=1.19, 95%CI=1.02-1.40, P=0.026), *Roseburia* (OR=1.20, 95%CI=1.03-1.41, P=0.018), *Burkholderiales* (OR=1.22, 95%CI=1.05-1.41, P=0.008) and *Terrisporobacter* (OR=1.25, 95%CI=1.04-1.49, P=0.016) had a potential causal effect on the increased risk of BD, whereas *Ruminiclostridium 5* (OR=0.86, 95%CI=0.75-0.98, P=0.028), *Candidatus Soleaferrea* (OR=0.91, 95%CI=0.84-0.98, P=0.020), *Victivallis* (OR=0.92, 95% CI=0.86-1.00, P=0.036) had a potential causal effect on the decreased risk of BD (**Figure 3**). The MR results of the Weighted Median, Weighted Mode, MR-Egger and MR-PRESSO methods were then used as additional validation of the IVW. Of the 13 taxa with solid MR results in IVW, four taxa (*Betaproteobacteria, Butyricimonas, Candidatus Soleaferrea* and *Burkholderiales*) showed MR-Egger results that were not parallel to those in IVW. Normally, in this case, we would need to re-examine the instrumental variables at more stringent thresholds (Chen et al., 2021). However, these four taxa already contain few instrumental variables, and it would be difficult to ensure the statistical power of the MR results if the number of instrumental variables were further reduced. 1 × 10^-5^ is the threshold chosen for most Mendelian randomization studies of the gut microbiota (Yu et al., 2023; Zeng et al., 2023). In addition, the causal effects of the IVW analysis were more precise than those of the MR-Egger analysis. Finally, this situation was already observed in the study by Luo et al. who ultimately chose to retain taxa for which the MR-Egger results did not parallel the IVW results (Luo et al., 2023). Thus, in the absence of heterogeneity and horizontal pleiotropy, it was ultimately acceptable to retain the IVW results for these four taxa.

### 3.3 Sensitivity analysis

**Figure 4:**
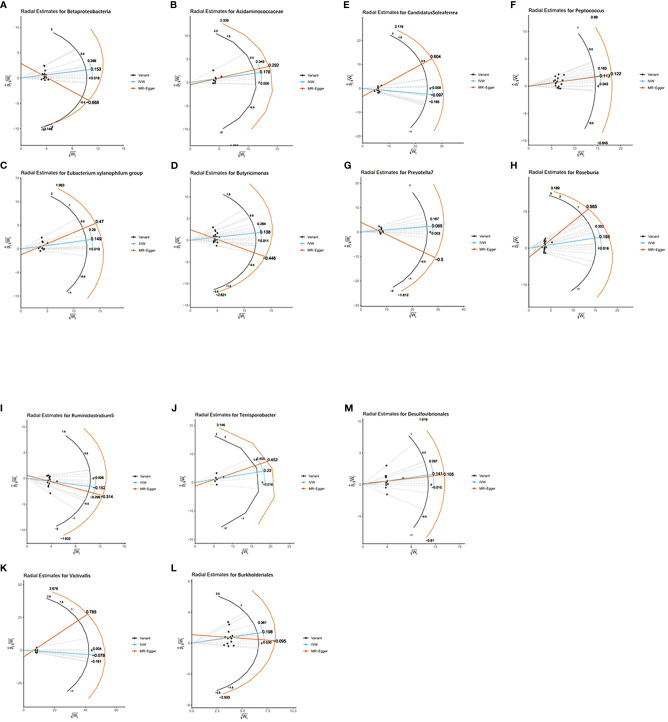
Radial plots of MR results for thirteen gut microbiota in bipolar disorder. **(A)** Radial estimate for Betaproteobacteria; **(B)** Radial estimate for Acidaminococcaceae; **(C)** Radial estimate for Eubacterium xylanophilum group; **(D)** Radial estimate for Butyricimonas; **(E)** Radial estimate for CandidatusSoleaferrea; **(F)** Radial estimate for Peptococcus; **(G)** Radial estimate for Prevotella 7; **(H)** Radial estimate for Roseburia; **(I)** Radial estimate for Ruminiclostridium 5; **(J)** Radial estimate for Terrisporobacter; **(K)** Radial estimate for Victivallis; **(L)** Radial estimate for Burkholderiales; **(M)** Radial estimate for Desulfovibrionales.

As MR results can be affected by invalid instrument bias or pleiotropy, this study examines the validity and robustness of the results through sensitivity analysis. With the exception of the *Ruminococcaceae NK4A214 group, Ruminococcaceae UCG-003* and *Bacillales*, none of the 13 gut microbial taxa were found to be horizontally pleiotropic in the MR-Egger intercept test and the MR-PRESSO global test (**Table 1**). **Figure 4** visually demonstrates that the MR results are not affected by horizontal pleiotropy. For these 13 taxa, the Cochran’s Q test and funnel plots provided no evidence for the presence of heterogeneity (**Figure S1**). In addition, the leave-one-out analysis demonstrates the robustness of the MR results, as the exclusion of any of the instrumental variables does not fundamentally affect the results (**Figure S2**). The visualisation results of the remaining analyses are shown in **Figures S3, 4**.

**Table 1:** Sensitivity analysis for 13 gut microbiota taxa associated with bipolar disorder.

id.exposure	Cochran’s Q	Cochran’s Q pval	Egger_intercept	Egger_intercept pval	MR-PRESSO global test pval
Betaproteobacteria	11.65	0.63	0.021	0.21	0.65
Acidaminococcaceae	5.77	0.57	-0.008	0.71	0.62
Eubacterium xylanophilum group	7.96	0.72	-0.010	0.56	0.74
Butyricimonas	21.49	0.16	0.017	0.35	0.19
Candidatus Soleaferrea	14.84	0.46	-0.030	0.09	0.47
Peptococcus	10.94	0.76	-0.001	0.95	0.77
Prevotella 7	9.17	0.52	-0.001	0.98	0.55
Roseburia	23.72	0.10	0.000	0.99	0.11
Ruminiclostridium 5	13.60	0.48	-0.002	0.88	0.50
Terrisporobacter	9.71	0.08	-0.002	0.95	0.16
Victivallis	13.21	0.28	-0.006	0.87	0.31
Burkholderiales	12.18	0.43	0.025	0.14	0.47
Desulfovibrionales	14.18	0.29	0.001	0.97	0.33

### 3.4 Replicated analysis after removing confounders-related Ivs

**Figure 5:**
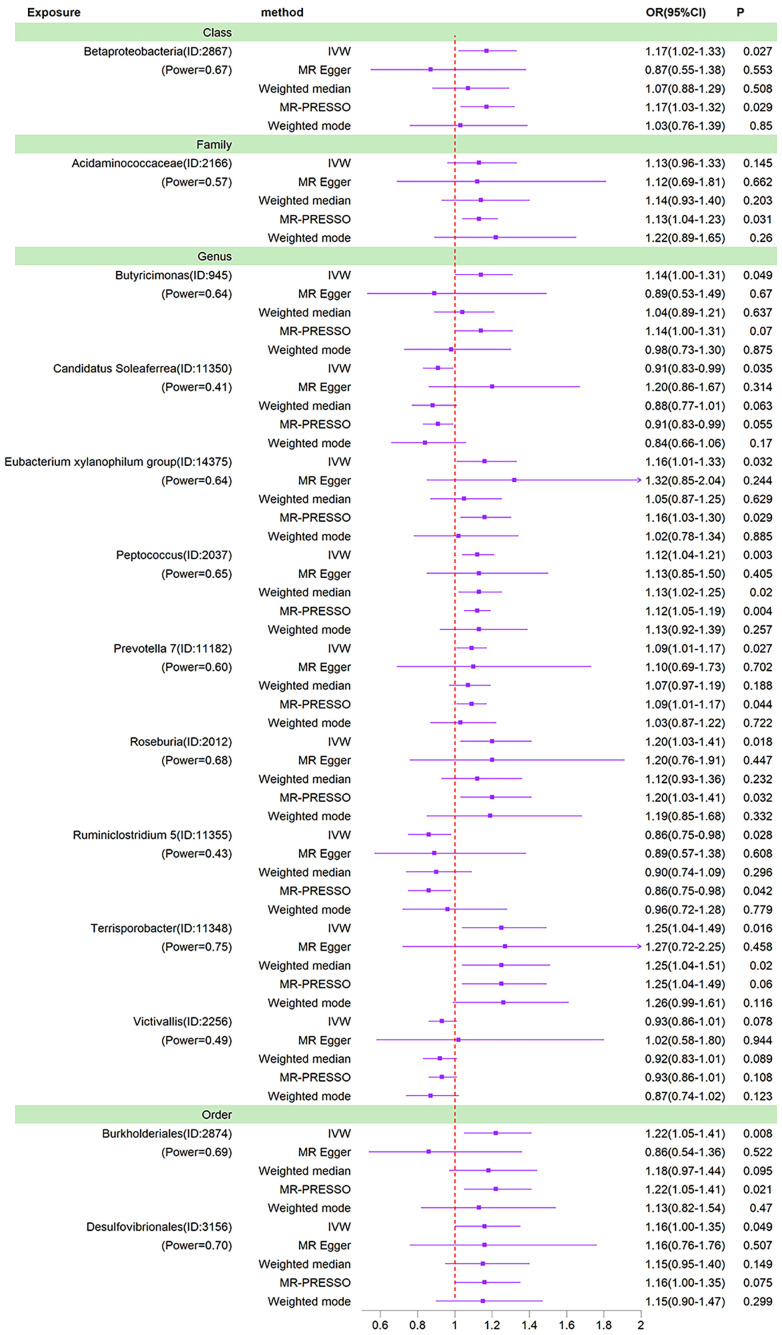
Forest plots of MR results for thirteen gut microbiota in bipolar disorder after removing confounding IVs. Method, statistical analysis methods; OR, odds ratio; 95%CI, 95% confidence interval; P, significance P-value; Power, the statistical power of causal effect estimates.

To further rule out confounding, we examined instrumental variables of 13 gut microbiota taxa associated with bipolar disorder using the PhenoScanner. Of these, rs6058181, rs113054641, rs72814525, rs12500231, rs56349194 were associated with obesity, rs45497800 with smoking and rs6494306 with type 2 diabetes. After removing these instrumental variables associated with confounders, the causal relationships between the 13 gut microbial taxa and BD were reassessed. The results showed that the causal relationships of the remaining gut microbial taxa remained significant, with the exception of *Acidaminococcaceae* and *Victivallis* (**Table S4**; **Figure 5**).

## 4 Discussion

After removing 15 unknown taxa, we used MR to analyse the causal relationship between the remaining 196 gut microbiota and bipolar disorder. Finally, we identified a total of 13 gut microbiota that were nominally causally related. Of these, *Betaproteobacteria, Acidaminococcaceae, Eubacterium xylanophilum group, Butyricimonas, Peptococcus, Prevotella 7, Roseburia, Terrisporobacter, Burkholderiales* and *Desulfovibrionales* had a potential causal effect on increased risk of BD, while *Candidatus Soleaferrea, Ruminiclostridium 5* and *Victivallis* had a potential causal effect on decreased risk of BD. The results suggest that the gut microbiota is both a potential marker for early identification of individuals at risk of BD and a target for optimal control strategies.

Many observational studies have found that changes in the gut microbiota are strongly associated with BD. A study by Vinberg et al. found that twins with bipolar disorder not only had reduced gut microbiota diversity compared to healthy twins, but also had significantly altered abundance of some gut microbiota (Vinberg et al., 2019). In a study of 234 cases of acute mania in bipolar disorder, antibiotic use was strongly associated with the induction and severity of acute mania in bipolar disorder (Yolken et al., 2016). Because of the limitations of observational studies, we conducted a large-scale systematic analysis of 196 gut microbiota taxa at the genetic level.

Our study identified 13 gut microbial taxa with nominal causal relationships, some of which were related to previous findings. For example, Lu et al. found significantly increased gut abundance of *Prevotella* in patients with BD compared to healthy controls, confirming the results of the present study (Lu et al., 2019). Hu et al. found significantly lower gut abundance of *Roseburia* in patients with BD compared to healthy controls, which contradicts the results of the present study (Hu et al., 2019). While some gut microbial taxa have been found to be associated with BD patients, such as a meta-analysis of 59 case-control studies that found an enrichment of *Actinobacteria, Oscillibacter, Megasphaera, Enterococcus* and a depletion of *Faecalibacterium* in BD patients, similar results were not found in the present study (Nikolova et al., 2021). This difference between genetically predicted and clinically observed outcomes may be due to the complex interactions between the gut microbiota. Further prospective randomised controlled trials are needed to validate this issue.

In recent years, as research has progressed, some evidence has emerged about the mechanisms by which the gut microbiota affect bipolar disorder. (i) Ecological dysbiosis of the microbiota leads to increased secretion of LPS and its entry into the somatic circulation. Once in the circulation, LPS binds to Toll-like receptor 4 (TLR4) and initiates inflammatory signalling leading to the release of a number of inflammatory factors such as IL-6 and IL-1 (Leblhuber et al., 2021). These inflammatory factors, which cross the blood-brain barrier and enter the brain tissue, can cause neuroinflammation, nerve damage and other manifestations that affect central function (Huang et al., 2021). (ii) The gut microbiota can directly or indirectly produce neurotransmitters (e.g. GABA, 5-HT, dopamine) that affect brain activity and may influence the development of BD by regulating systemic and central neurotransmitter concentrations. In addition, as the vagus nerve has sensory afferent functions, gut microbiota may influence the activity of brain functions by activating the vagus nerve (Gondalia et al., 2019). (iii) Gut microbiota may influence episodes of bipolar disorder by activating the hypothalamic-pituitary-adrenal (HPA) axis and modulating levels of brain-derived neurotrophic factor (BDNF) (Sudo et al., 2004; Maqsood and Stone, 2016). (iv) Gut microbiota can influence the uptake and utilisation of tryptophan, a key signalling molecule in the gut-brain axis involved in mood regulation (Asan et al., 2013; O'Mahony et al., 2015; Sun et al., 2019).

Although our results found a causal association between gut microbiota and bipolar disorder, our study has some limitations. First, MR results may be biased if sample overlap occurs in Mendelian randomisation studies. In the present study, although we could not explicitly calculate the sample overlap rate, the GWAS summary statistics of gut microbiota and bipolar disorder were obtained from completely different databases, and it is unlikely that sample overlap occurred. In addition, as the GWAS data did not provide detailed individual information, we were unable to perform further subgroup analyses based on factors such as gender and age. Although there are some limitations to this study, a number of sensitivity analyses have confirmed the robustness and reliability of the findings, and therefore this study is valuable.

## 5 Conclusion

The present study demonstrated a causal relationship between gut microbiota and bipolar disorder using MR analysis. Specifically, *Betaproteobacteria, Acidaminococcaceae, Eubacterium xylanophilum group, Butyricimonas, Peptococcus, Prevotella 7, Roseburia, Terrisporobacter, Burkholderiales* and *Desulfovibrionales* increased the risk of BD, while *Candidatus Soleaferrea, Ruminiclostridium 5 and Victivallis* decreased the risk of BD. The greatest value of our research is to provide new biomarkers and potential therapeutic targets for the prevention and treatment of BD.

## Data availability statement

Publicly available datasets were analyzed in this study. This data can be found here: https://mibiogen.gcc.rug.nl https://gwas.mrcieu.ac.uk/.

## Author contributions

RX and X-JW designed the study, contributed to the data analysis, and wrote the manuscript. SL, L-YL and YZ contributed to the data analysis and data interpretation. B-QF and G-CL contributed to manuscript writing and revision of the manuscript. All authors contributed to the article and approved the submitted version.
