# Supplementary material for: Causal effects of gut microbiota on the risk of bipolar disorder: a Mendelian randomization study
Source: Front Microbiomes. 2023 Sep 21;2:1249518. doi: 10.3389/frmbi.2023.1249518 (PMC12993656; doi:10.3389/frmbi.2023.1249518)
Supplement: Supplementary file 1 [file DataSheet_1.zip › Supplementary Figures.DOCX]

Supplementary Material

Causal effects of gut microbiota on the risk of bipolar disorder: a Mendelian randomization study

Ran Xu^1^, Shuo Liu^1^, Lu-yi Li^1^, Yin Zhang^2^, Guang-cheng Luo^1，2^ , Bo-qin Fang^1^, Xin-jun Wang^1，2^*

*Corresponding author: Xinjun Wang，Department of Urology，Zhongshan Hospital Xiamen University. No. 201-209, Hubin South Road, Siming District, Xiamen, Fujian, 361000, China. Tel:86-0592-2292201. Fax:86-0592-2292201. E-mail: wxj@xmu.edu.cn.


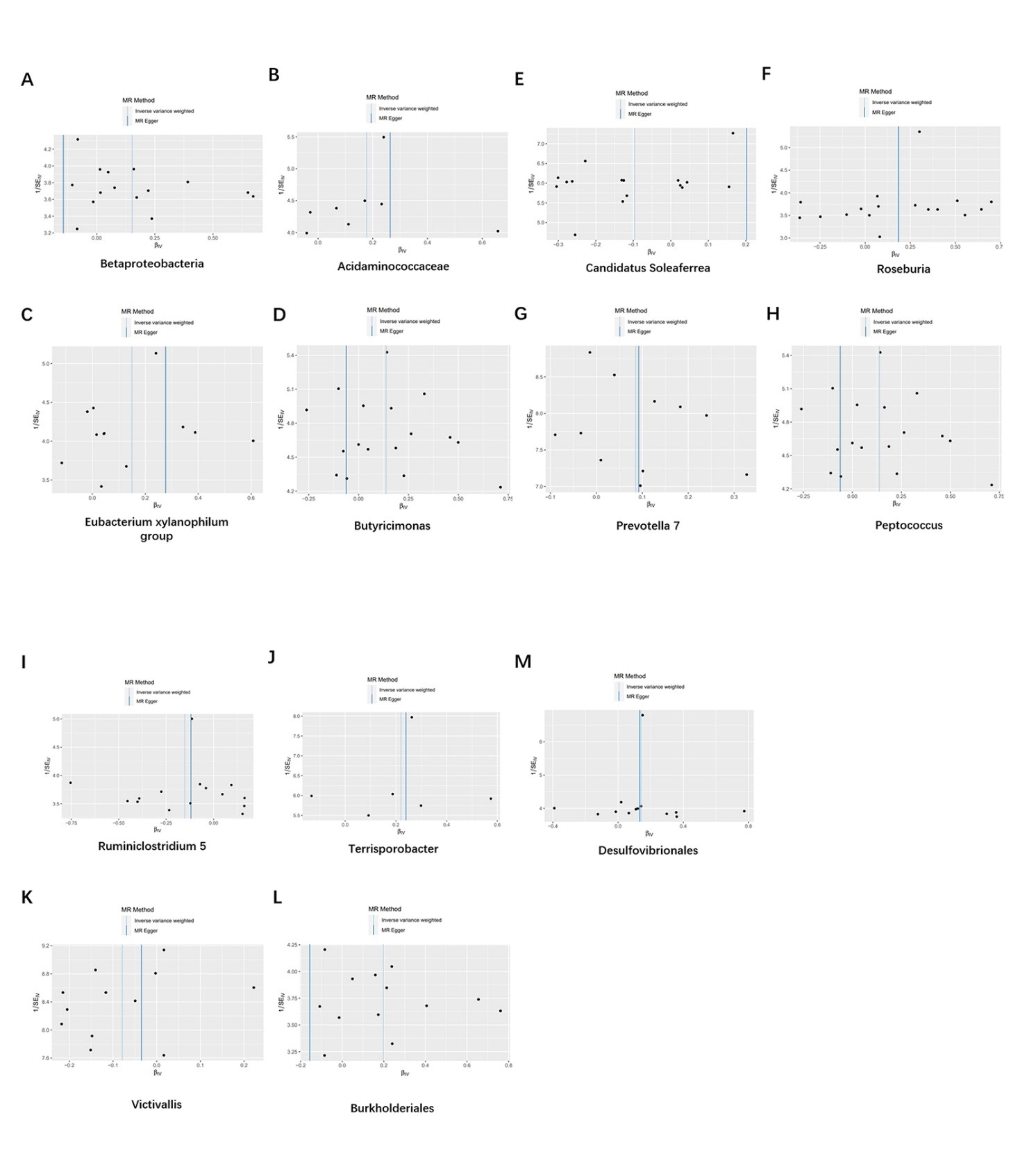


**Figure S1:** Funnel plots from 13 gut microbiota taxa on the risk of BD.


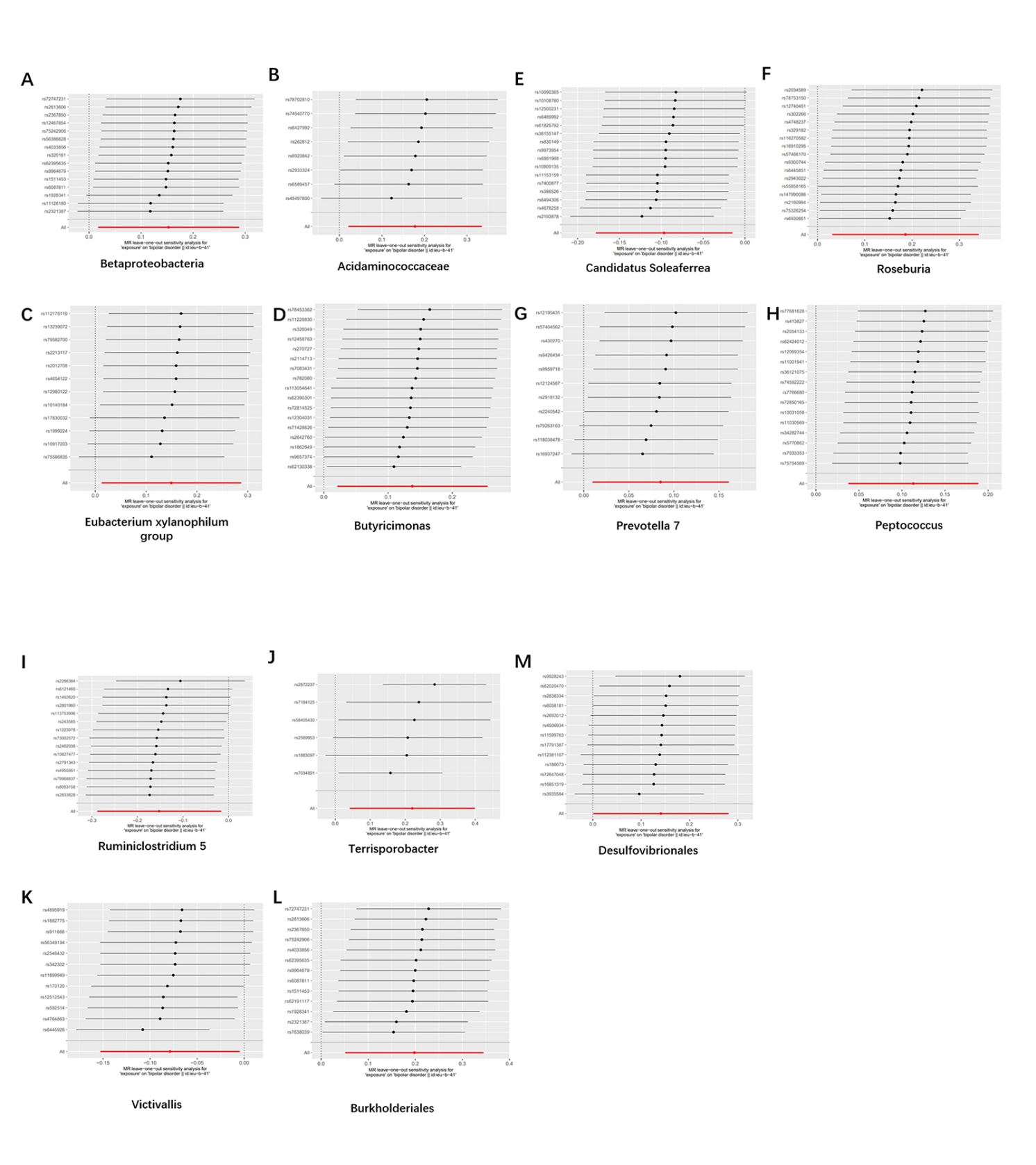


**Figure S2:** Leave-one-out analysis from 13 gut microbiota taxa on the risk of BD.

**
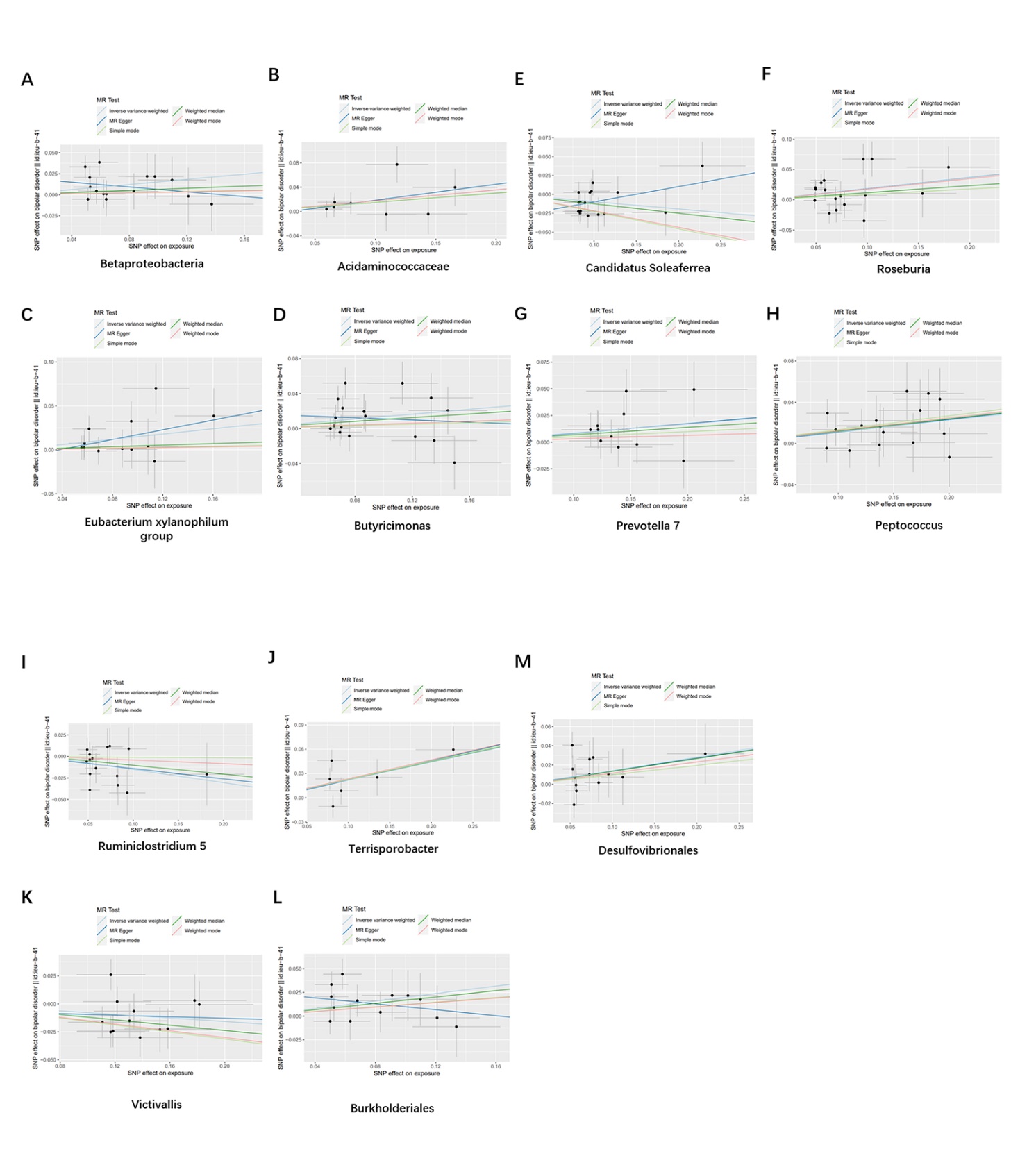
**

**Figure S3:** Scatter plot from 13 gut microbiota taxa on the risk of BD.


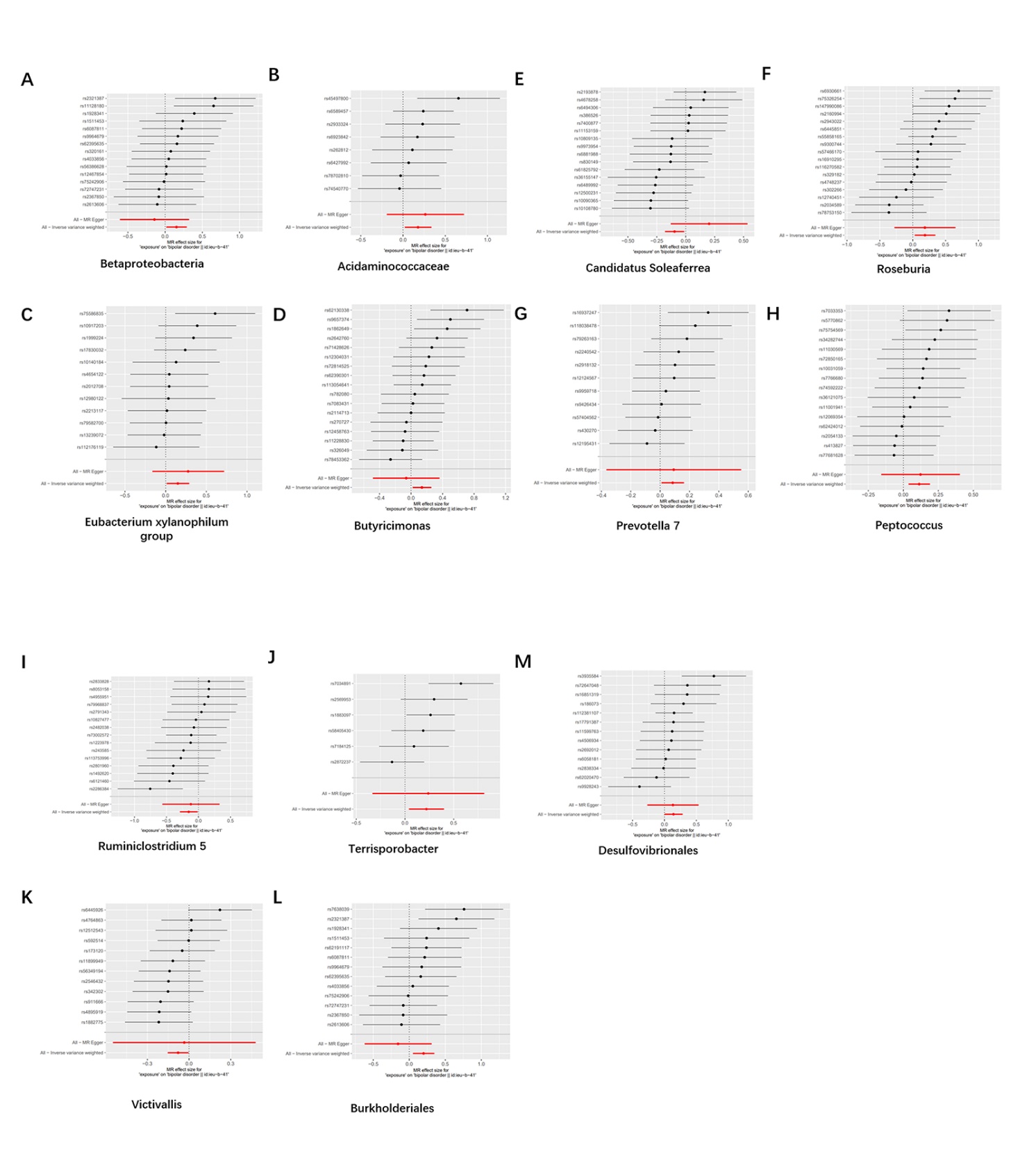


**Figure S4:** Forest plot from 13 gut microbiota taxa on the risk of BD.
